# Comparative Anatomical Analysis of Bark Structure in 10 *Quercus* Species

**DOI:** 10.3390/plants13131871

**Published:** 2024-07-06

**Authors:** Changzhao Li, Xiaorui Yang, Songyang Chen, Yuxi Huang, Yushan Yang, Jian Qiu

**Affiliations:** 1 Yunnan Provincial Key Laboratory of Wood Adhesives and Glued Products, Southwest Forestry University, Kunming 650224, China; lichangzhao1325@163.com (C.L.); xiaorui980115@163.com (X.Y.); 15264202895@163.com (Y.H.); ysyoung@swfu.edu.cn (Y.Y.); 2 Tsingyan Lingzhi Information Consulting (Beijing) Co., Ltd., Beijing 100088, China; c936896186@163.com; 3 International Joint Research Center for Biomass Materials, Southwest Forestry University, Kunming 650224, China

**Keywords:** *Quercus* species, bark anatomy, microscopy, phloem structure

## Abstract

Detailed anatomical features of bark are used and interpreted in plant taxonomy, phylogenetics, and other areas of plant science. However, the delicate nature of bark cells, combined with the difficulty of obtaining high-quality sections and reliable data, limits the potential for utilizing and processing bark. In this study, the anatomical structure of the bark of 10 *Quercus* species growing in Yunnan Province, China, was characterized in detail. The results indicate that the anatomical features of the barks of 10 *Quercus* spp. show a certain degree of consistency. Specifically, sieve tubes are distributed in solitary elements or in small groups, mostly as compound sieve plates containing 2–8 sieve areas, suggesting that *Quercus* spp. may occupy a conservative evolutionary position. Additionally, for the first time, this study reports the presence of simple sieve plates in the sieve tube elements of *Quercus* phloem. Each sieve tube element has a companion cell on one side. The companion cell strands contain 2–7 cells. Axial parenchyma is diffuse, with parenchyma strands typically consisting of 4–7 cells; druses are present within chambered crystalliferous cells. Phloem rays are of two distinct sizes and often exhibit dilatation and sclerification, and the ray composition consists of procumbent cells. Sclerenchyma is composed of fibers and sclereids, both of which contain prismatic crystals. Most of the fibers are gelatinous fibers, which are distributed in discontinuous tangential bands of about five cells in width. Sclereids appear in clusters. The presence of sclerenchyma provides mechanical support to the bark, reducing the collapse of the phloem. Periderm usually consists of around 10–30 layers of phellem, and *Quercus acutissima* and *Q. variabilis* can reach dozens or hundreds layers. The phelloderm typically consists of from two to five layers, with *Q. variabilis* having up to ten or more layers. The filling tissue of lenticels in all *Quercus* species is nonstratified (homogeneous) and largely nonsuberized. Overall, this study enriches our comprehension of *Quercus* bark anatomy, elucidating evolutionary patterns, functional adaptations, and ecological ramifications within this significant botanical genus.

## 1. Introduction

The bark refers to all tissues produced outside the vascular cambium of trees, shrubs, or lianas. It comprises two distinct layers: the inner bark (phloem), typically lighter in color, and the outer bark (rhytidome), usually darker [1,2,3]. In the early stages of tree growth, the bark is composed of primary tissues, primarily including the epidermis, cortex, and primary phloem. While these tissues may lose functionality and undergo deformation or gradual abscission shortly after their formation, certain tree species sustain them over prolonged periods. The enduring constituents of bark primarily constitute secondary tissues, including the secondary phloem and periderm [1,2,4,5,6].

The division of various cells in the secondary phloem mirrors that of the secondary xylem, exhibiting distinct axial and radial systems. The axial system predominantly comprises sieve elements responsible for conduction, thick-walled tissues provide support, and axial parenchyma tissues are tasked with storage and transport. Meanwhile, the radial system is composed of thin-walled ray parenchyma tissues, which have storage and transport functions [4,5]. Variations in cell type, abundance, morphology, and arrangement within these broad tissues serve as rich subjects of phylogenetic analyses [7]. These variations are likely to influence the patterns of plant diversification.

Given the diverse array of bark characteristics, the initial step in developing effective methods for utilizing and processing bark is to conduct an in-depth structural analysis. Despite the extensive historical exploration facilitated by microscopy, research on bark lags behind that of wood (secondary xylem) [1,5], primarily due to technological limitations.

In wood specimens, the bark that remains attached is typically readily available, allowing for the collection of a substantial amount of bark samples in a short period. Due to the delicate nature of bark cells, these cells will undergo collapse or degradation upon drying, resulting in an inability to provide high-quality sections and reliable data. This limitation necessitates the acquisition of fresh samples that are promptly fixed with a liquid fixative and preserved. These materials are then stored in alcohol or suitable fixatives to maintain numerous intact cells exhibiting exceptional detail, including sieve plates and lateral sieve areas. Larger samples (exceeding 1 cm^3^) can be effectively fixed with formaldehyde–acetic acid (FAA), while smaller ones can be treated with paraformaldehyde, CRAFF III/IV/V, or glutaraldehyde for preservation [8,9,10,11].

Wood contains cells with durable and rigid cell walls. Bark, being a heterogeneous plant part, consists of both soft nonlignified cells and hard lignified cells. To address this characteristic, traditional methods typically involve the use of hydrofluoric acid (HF) [8] for softening, but this is highly toxic and corrosive. Ethylenediamine can induce cell wall expansion, reducing its rigidity, especially in thick-walled portions. This effect enhances the uniformity of heterogeneous plant tissues, facilitating subsequent slicing processes [12]. Nevertheless, not all bark samples require softening, as this process is unnecessary for materials that are non-heterogeneous or relatively soft [13].

Embedding bark in a matrix is beneficial for sectioning, as it allows the embedding medium to be evenly distributed within the sample, ensuring its integration and stability during slicing. Previously, high-quality bark sections were predominantly prepared using celloidin (parlodion) [8,10,14,15]. Nevertheless, due to the challenges associated with its preparation and its flammability and explosiveness, celloidin has become less readily available and is gradually being phased out from teaching and research applications. Polyethylene glycol (PEG) 1500 [16] exhibits excellent permeability and is particularly effective for embedding larger samples or even entire stems [17,18]. As a water-soluble embedding agent, it can be easily removed during the sectioning process, reducing the reliance on organic chemicals and minimizing potential environmental and experimental risks. The use of polystyrene foam resin [6] reduces the risk of sample breakage during sectioning and in subsequent steps. Although this method may slow down the sectioning process, it ensures that higher-quality sections are obtained.

Aiming to determine the anatomical characteristics of wood, the IAWA hardwood and softwood lists [19,20] were successively published by the International Association of Wood Anatomists (IAWA) at the end of the last century. These lists, commonly referred to as the IAWA lists, have played a crucial role in wood identification by establishing the uniform recording and description of anatomical features across different wood types through standardized coding systems. This standardization has significantly facilitated the development of web-based retrieval databases such as Inside Wood [21] and EyeWood [22], which provide researchers with comparable data, abundant resources, and references. Furthermore, in 2014, IAWA expanded its scope by publishing a list specifically focusing on bark anatomy [1]. This new initiative not only advanced the application and dissemination of bark anatomical research but also presented an opportunity for the establishment of a bark retrieval database.

*Quercus* L. is a major woody genus in the northern hemisphere, notably present in North America, Europe, and particularly in Eastern Asia, comprising approximately 500 species of trees and shrubs [23,24]. The bark of oak (*Quercus* spp.) has been traditionally used in folk medicine for centuries due to its medicinal properties, which have shown potential in treating various diseases. Throughout diverse geographic regions, indigenous populations historically utilized oak bark for its medicinal properties [24]. The bark of the oak tree is highly esteemed for its therapeutic value, particularly in the form of boiled extracts. These boiled extracts exhibit anti-inflammatory, antibacterial, and antihemorrhagic properties, making them potent antiseptic and hemostatic agents [25] that are commonly applied to alleviate toothache and address gastropathies. Additionally, oak bark is employed as a remedy for burn injuries [24,26], and has been suggested for the treatment of patients with high levels of blood sugar and for the treatment of sore throat [25,27].

The bark of *Quercus* species, notably *Q. suber*, holds significant value as a resource, primarily owing to its thick (cork) layer, rendering it well-suited for sustainable cork production with notable economic implications [28]. In contemporary industries, cork demonstrates remarkable versatility and widespread utility. Principally, it serves as the primary material for the production of natural cork stoppers, contributing over 15 billion stoppers annually to the wine industry. Furthermore, cork tiles, valued for their durability and resistance to moisture, are extensively employed in flooring and wall paneling applications. Its exceptional thermal insulation properties render it indispensable for roofing, wall cladding, and flooring solutions in both residential and industrial contexts. Cork’s exceptional acoustical properties make it a preferred material for noise reduction across diverse environments, ranging from conference rooms to theaters. Moreover, it finds utility in concrete structures, forming expansion joint fillers and anti-vibration layers. In the realm of sports, cork is utilized in the fabrication of various equipment, including hockey balls and baseball bats. Beyond industrial and sporting applications, cork finds versatile usage in the creation of gift items, novelty products, and contemporary furniture designs. Lastly, architects and designers have explored the aesthetic allure of cork in innovative architectural embellishments and decor [29,30].

This study was designed to investigate the bark anatomical features of 10 species within the *Quercus*, with the objective of gaining a comprehensive understanding of their anatomical structure and function. Through a systematic examination of bark tissue organization, cell types, and cell arrangement, this research seeks to elucidate the similarities and differences among these *Quercus* species and explore potential correlations among these features and their evolutionary relationships. The ultimate objective is to provide scientific evidence and a reference for plant classification and biodiversity conservation.

## 2. Results

### 2.1. Sieve Tubes and Companion Cells

**Figure 1:**
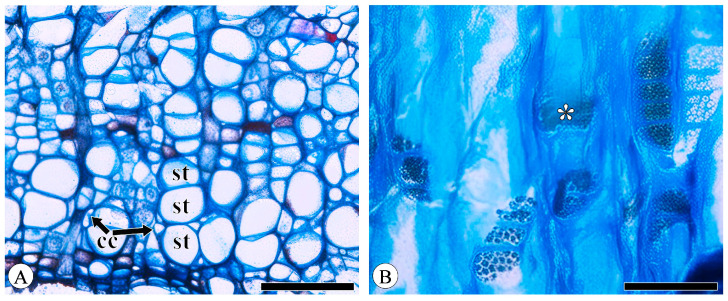
Sieve tubes and companion cells. (**A**) Sieve tubes (st) in solitary and in small groups or in radial rows, with one companion cell (cc) per sieve tube, *Q. variabilis*, transverse section; (**B**) compound sieve plate and simple sieve plate (asterisk), *Q. variabilis*, radial section. Scale bar for (**A**) = 100 μm; (**B**) = 50 μm.

**Figure 2:**
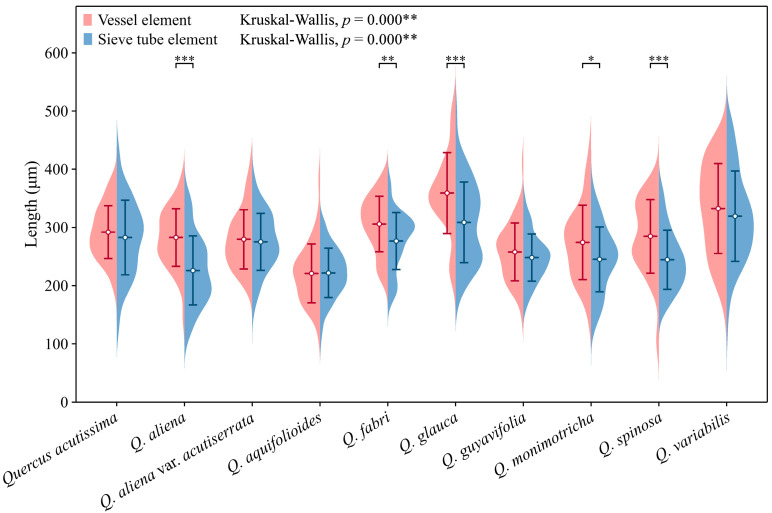
Sieve tube element and vessel element length. Sieve tube element: *Q. acutissima*: 283 ± 64 μm; *Q. aliena*: 226 ± 59 μm; *Q. aliena* var. *acutiserrata*: 275 ± 49 μm; *Q. aquifolioides*: 222 ± 43 μm; *Q. fabri*: 277 ± 49 μm; *Q. glauca*: 309 ± 69 μm; *Q. guyavifolia*: 248 ± 40 μm; *Q. monimotricha*: 245 ± 56 μm; *Q. spinosa*: 245 ± 51 μm; *Q. variabilis*: 319 ± 78 μm. Vessel element: *Q. acutissima*: 292 ± 45 μm; *Q. aliena*: 283 ± 50 μm; *Q. aliena* var. *acutiserrata*: 280 ± 51 μm; *Q. aquifolioides*: 221 ± 51 μm; *Q. fabri*: 306 ± 48 μm; *Q. glauca*: 359 ± 70 μm; *Q. guyavifolia*: 258 ± 50 μm; *Q. monimotricha*: 274 ± 64 μm; *Q. spinosa*: 285 ± 63 μm; *Q. variabilis*: 333 ± 77 μm. *: *p* < 0.05; **: *p* < 0.01; ***: *p* < 0.001.

**Figure 3:**
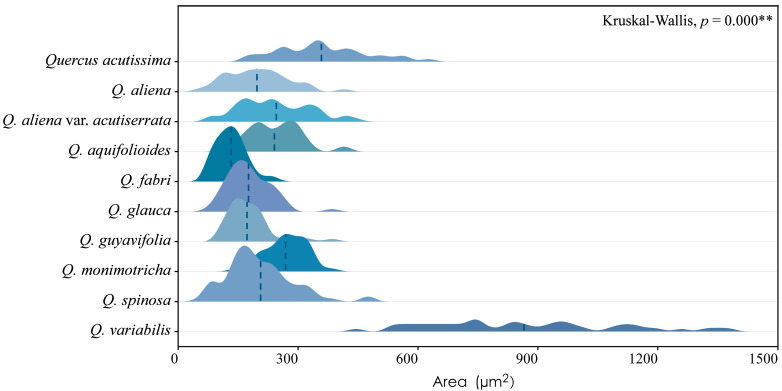
Sieve tube area. *Q. acutissima*: 361 ± 109 μm^2^; *Q. aliena*: 198 ± 74 μm^2^; *Q. aliena* var. *acutiserrata*: 245 ± 88 μm^2^; *Q. aquifolioides*: 242 ± 65 μm^2^; *Q. fabri*: 134 ± 36 μm^2^; *Q. glauca*: 178 ± 52 μm^2^; *Q. guyavifolia*: 176 ± 56 μm^2^; *Q. monimotricha*: 270 ± 51 μm^2^; *Q. spinosa*: 204 ± 83 μm^2^; *Q. variabilis*: 867 ± 235 μm^2^. **: *p* < 0.01.

Sieve tube elements, characterized by sieve plates with wide pores and lateral sieve areas with narrow pores, are interconnected in series to form sieve tubes. The experimental results reveal that these sieve tubes are predominantly solitary or form small groups, and are occasionally arranged in radial rows due to the interruption caused by phloem rays (Figure 1A). In the conducting phloem, the sieve tubes in the transverse section appear nearly circular to irregular in shape, with thin cell walls that are nonlignified, and are often confounded with axial parenchyma. The sieve plates are compound, scalariform and variably deposited with callose, with 2–8 sieve areas per plate (Figure 1B). Some scalariform sieve plates are not densely arranged, with certain distances between individual sieve areas. Significantly, a limited number of simple sieve plates were also identified in 10 species (Figure 1B and Figure S1), with the frequency of simple sieve plates being consistently less than 5%. Detailed statistical data on the frequency of simple sieve plates can be found in Table S1. The sieve tube elements may appear with one end as a simple sieve plate and the other end as a compound sieve plate. The lateral sieve areas are prominent and large, scalariform. The length of sieve tube elements ranges between 200 and 350 μm, comparable to or shorter than vessel elements. Mann–Whitney U tests revealed significant differences in vessel and sieve tube lengths among the species, with *Q. aliena* (*p* < 0.001), *Q. fabri* (*p* < 0.01), *Q. glauca* (*p* < 0.001), *Q. monimotricha* (*p* < 0.05), and *Q. spinosa* (*p* < 0.001) showing notable differences, while other species did not exhibit significant differences. Additionally, Kruskal–Wallis tests were performed separately for vessel and sieve tube lengths, with both yielding significant results (*p* < 0.001), indicating overall significant differences among the species (Figure 2). Detailed data are presented in Table S2. Transverse section measurements reveal that the average sieve tube area of *Q. variabilis* is 867 μm^2^, that of *Q. acutissima* is 366 μm^2^, and the remaining species range between 150 and 300 μm^2^. a Kruskal–Wallis test conducted on the sieve tube area for the 10 species showed significant differences (*p* < 0.001), indicating that the sieve tube area varies significantly across species (Figure 3). The detailed measurement and analysis data are presented in Table S3.

**Figure 4:**
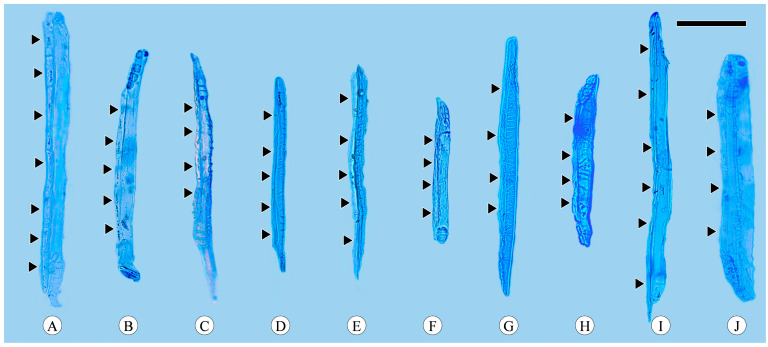
Sieve tube elements with associated companion cells (arrows). (**A**) Companion cells in strands of seven cells, *Quercus acutissima*; (**B**) companion cells in strands of five cells, *Q. aliena*; (**C**) companion cells in strands of four cells, *Q. aliena* var. *acutiserrata*; (**D**) companion cells in strands of five cells, *Q. aquifolioides*; (**E**) companion cells in strands of five cells, *Q. fabri*; (**F**) companion cells in strands of four cells, *Q. glauca*; (**G**) companion cells in strands of four cells, *Q. guyavifolia*; (**H**) companion cells in strands of four cells, *Q. monimotricha*; (**I**) companion cells in strands of six cells, *Q. spinosa*; (**J**) companion cells in strands of four cells, *Q. variabilis*. Scale bar = 100 μm.

Companion cells are a distinctive feature of the phloem in angiosperms. They originate from the same mother cell as sieve tube elements, to which they are ontogenetically and functionally related. In the transverse section, only one companion cell is typically found lying on the side of a sieve tube element (Figure 1A), and it is difficult to observe companion cells in longitudinal sections, including radial and tangential sections. The companion cell strands can be clearly identified based on the macerated sieve tube. These strands are equivalent in length to the sieve tube elements, with occasional instances where one end of a companion cell strand slightly protrudes beyond the sieve tube element. Companion cells typically form strands consisting of from two to seven cells (Figure 4), and longer sieve tube elements usually have more associated companion cells.

The conducting phloem is also referred to as the functioning phloem. Within the secondary phloem of angiosperms, it is identified by the presence of living sieve tubes accompanied by companion cells adjacent to the cambium. The sieve elements of nonconducting phloem lack a conduction function and are prone to collapse. In all samples, it is challenging to delineate the boundary between a conducting phloem and a nonconducting phloem. Due to the mixed arrangement of sieve tubes and axial parenchyma, it is also difficult to observe the evident compression of collapsed sieve tubes in the nonconducting phloem.

### 2.2. Axial Parenchyma

**Figure 5:**
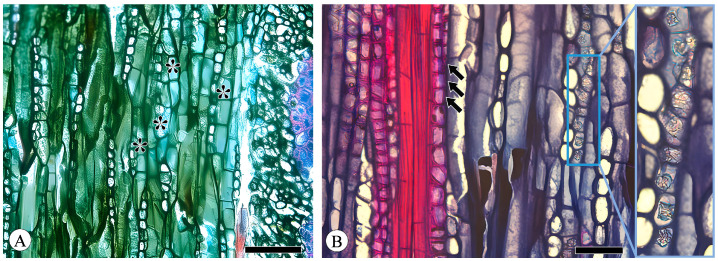
Axial parenchyma. (**A**) Axial parenchyma strands (asterisk), *Q*. *acutissima*, tangential section; (**B**) Druses located in the chambered axial parenchyma cells, with prismatic crystals (arrows) in fibers, *Q. aquifolioides*, tangential section. Scale bar for (**A**) = 100 μm; (**B**) = 50 μm.

Axial parenchyma strands typically consist of from four to seven cells (Figure 5A), except for crystalliferous cells, and are approximately equal to or slightly longer than the sieve tube elements in terms of length. Crystals are located in the chambered axial parenchyma cells, with the chambers numbering up to several dozen, and the crystal shape is predominantly druses (Figure 5B). In certain crystal-bearing strands, individual cells undergo additional subdivision through the formation of horizontal and sometimes vertical walls before crystallization takes place. As a result, these strands exhibit a higher number of cells compared to the typical parenchyma strands found within the same tissue. Crystalliferous axial parenchyma is frequently positioned at the periphery of sclerenchyma bands.

### 2.3. Rays

**Figure 6:**
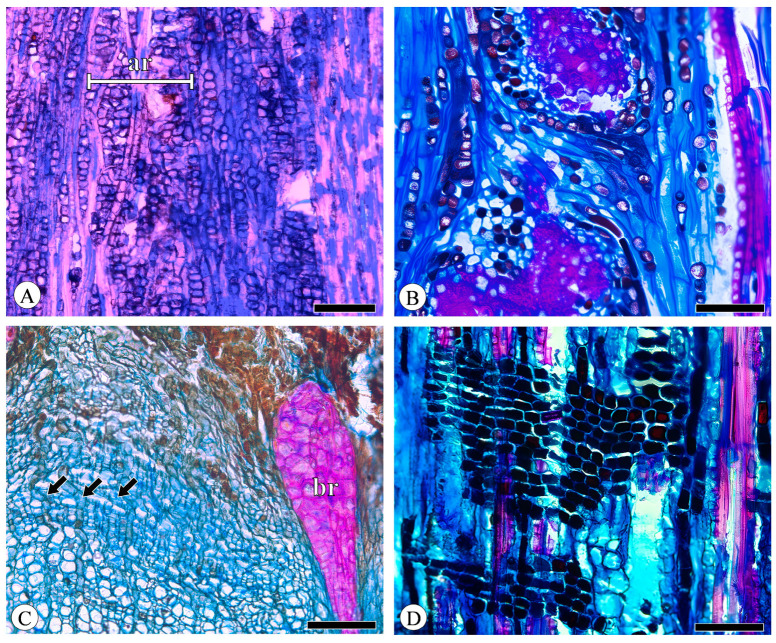
Rays. (**A**) Aggregate rays (ar) composed of narrow rays, *Q. aquifolioides*, tangential section; (**B**) aggregate rays composed of broad rays, *Q. acutissima*, tangential section; (**C**) broad rays (br) undergo sclerification and dilatation, whereas uniseriate rays (arrows) do not, *Q. variabilis*, transverse section; (**D**) all ray cells procumbent, *Q. fabri*, radial section. Scale bar for (**A**) = 300 μm; (**B**,**D**) = 100 μm; (**C**) = 200 μm.

The phloem rays consist of a cluster of parenchyma cells, demonstrating variability in both height and width. These cells originate from the ray initials within the vascular cambium and extend radially into the secondary phloem. All the detected species of phloem rays are exclusively uniseriate. *Q. aquifolioides* exhibits aggregate rays, which are composed of narrow rays (Figure 6A). While the other species have broad rays, commonly more than 10-seriate and exceeding 1 mm in height, *Q. acutissima* exhibits broad rays that are so closely associated with one another that they form aggregate rays (Figure 6B). In broad rays, almost all ray cells undergo sclerification to form groups of sclereids (Figure 6B,C), and a phenomenon closes the cambium. Dilatation refers to the expansion of bark circumference through the division and expansion of parenchyma cells to accommodate secondary xylem growth. The broad rays also undergo significant dilatation, which is particularly evident in *Q. acutissima* and *Q. variabilis*, being relatively narrow near the cambium and extremely wide towards the outer bark and appearing wedge-shaped (Figure 6C). The uniseriate rays do not undergo sclerification, even extending into the cortex, but due to cell collapse, the course of the rays exhibits slight undulations and no dilatation or very weak dilatation occurs (Figure 6C). Ray composition is uniformly procumbent (Figure 6D).

**Figure 7:**
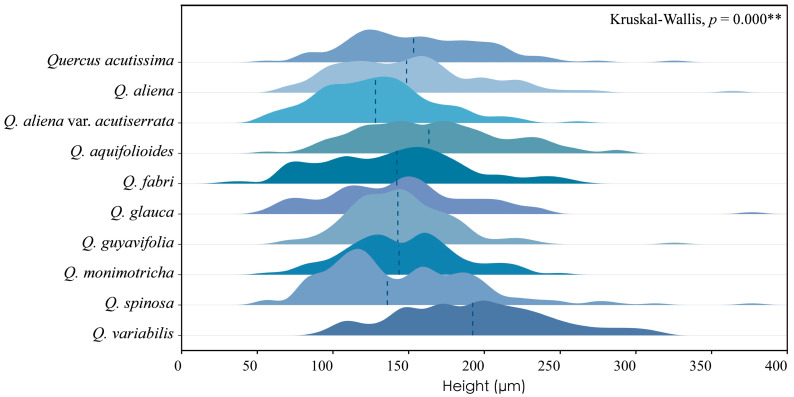
Ray height. *Q. acutissima*: 157 ± 45 μm; *Q. aliena*: 150 ± 48 μm; *Q. aliena* var. *acutiserrata*: 130 ± 38 μm; *Q. aquifolioides*: 166 ± 47 μm; *Q. fabri*: 140 ± 48 μm; *Q. glauca*: 144 ± 49 μm; *Q. guyavifolia*: 145 ± 35 μm; *Q. monimotricha*: 146 ± 37 μm; *Q. spinosa*: 146 ± 51 μm; *Q. variabilis*: 193 ± 50 μm. **: *p* < 0.01.

Across species, the height of phloem rays exhibits significant differences, as shown by Kruskal–Wallis tests (*p* < 0.001), underscoring the substantial variability among species. Despite these differences, phloem ray heights consistently fall within the range of 100 to 200 μm, indicating a stable characteristic that may serve as a valuable diagnostic feature within this taxon (Figure 7). The detailed measurement and analysis data are presented in Table S4. *: *p* < 0.05; **: *p* < 0.01; ***: *p* < 0.001.

### 2.4. Sclerenchyma

**Figure 8:**
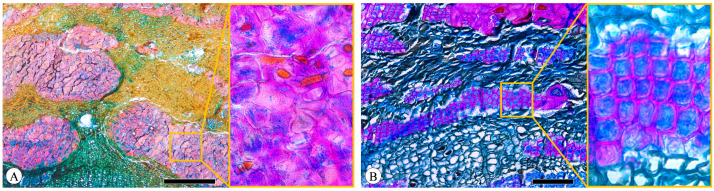
Sclerenchyma. (**A**) Sclereids in clusters, *Q. acutissima*, transverse section; (**B**) fibers in tangential bands, *Q. spinosa*, transverse section. Scale bar for (**A**) = 500 μm; (**B**) = 100 μm.

Sclerenchyma is a tissue composed of sclerified cells with diverse shapes and sizes, with secondary walls that are typically lignified. In the examined phloem, sclereids predominantly appeared in clusters (Figure 8A), and phloem fibers were arranged discontinuously in tangential bands approximately five cells wide (Figure 8B). These bands alternate with those of sieve tubes, companion cells, and parenchyma cells. The phloem fibers contain numerous crystals, predominantly prismatic and often numbering several dozen (Figure 5B). While the stone cells are generally uniform in diameter but vary in size, they also frequently contain prismatic crystals. In *Q. acutissima* and *Q. variabilis*, the sclerenchyma cell bands are predominantly composed of sclereids. No fiber-sclereids were observed. Septate phloem fibers were not detected.

**Figure 9:**
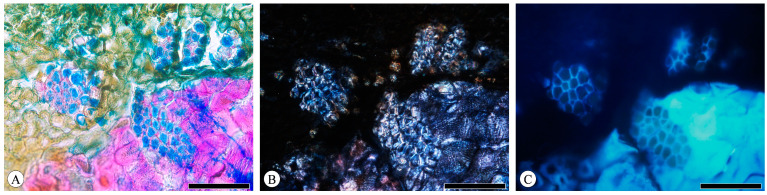
Gelatinous fibers in *Q. acutissima*, transverse section. (**A**) Brightfield microscopy; (**B**) polarized light microscopy; (**C**) fluorescence microscopy. Scale bar for (**A**–**C**) = 100 μm.

The majority of the observed phloem fibers are gelatinous fibers (G-fibers), which bear similarities to tension wood fibers in the xylem. The innermost thick secondary wall (also called the G-layer) demonstrates a distinct layering from the outer wall. Under brightfield microscopy, the outer wall of G-fibers can be stained red with safranin, while the inner layer is stained blue with astra-blue (Figure 9A). The presence of a crystalliferous region of cellulose within the cell wall can be qualitatively determined through polarized light observation, thus enabling the assessment of cellulose distribution. The phenolic compounds present in plant cell walls exhibit fluorescence when excited by light, attributed to the lignin content in plants. Lignin concentration in the cell wall can be determined based on autofluorescence intensity. Given its high cellulose content and low or absent lignin content, the G-layer exhibits a birefringent brightening phenomenon under polarized light, with consistent brightness across both layers (Figure 9B). Fluorescence is more pronounced on the outer wall compared to the inner one (Figure 9C).

**Figure 10:**
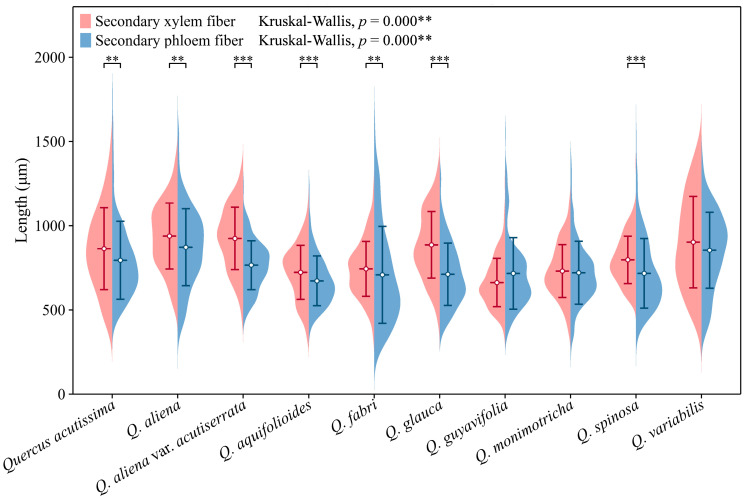
Secondary phloem and xylem fiber length. Secondary phloem fiber: *Q. acutissima*: 794 ± 231 μm; *Q. aliena*: 872 ± 228 μm; *Q. aliena* var. *acutiserrata*: 766 ± 145 μm; *Q. aquifolioides*: 673 ± 148 μm; *Q. fabri*: 708 ± 288 μm; *Q. glauca*: 712 ± 185 μm; *Q. guyavifolia*: 717 ± 213 μm; *Q. monimotricha*: 721 ± 187 μm; *Q. spinosa*: 717 ± 207 μm; *Q. variabilis*: 854 ± 225 μm. Secondary xylem fiber: *Q. acutissima*: 864 ± 243 μm; *Q. aliena*: 939 ± 196 μm; *Q. aliena* var. *acutiserrata*: 924 ± 185 μm; *Q. aquifolioides*: 723 ± 161 μm; *Q. fabri*: 744 ± 163 μm; *Q. glauca*: 886 ± 197 μm; *Q. guyavifolia*: 674 ± 147 μm; *Q. monimotricha*: 729 ± 158 μm; *Q. spinosa*: 797 ± 141 μm; *Q. variabilis*: 903 ± 271 μm. **: *p* < 0.01; ***: *p* < 0.001.

The lengths of secondary phloem fibers in each species fall within the range of approximately 650 to 850 μm. With the exception of *Q. guyavifolia*, whose secondary phloem fibers are slightly longer than those of secondary xylem fibers, the remaining species exhibit the opposite phenomenon. Mann–Whitney U tests conducted on secondary phloem and xylem fiber lengths across species revealed significant differences. Specifically, *Q. acutissima* (*p* < 0.01), *Q. aliena* (*p* < 0.01), *Q. aliena* var. *acutiserrata* (*p* < 0.001), *Q. aquifolioides* (*p* < 0.001), *Q. fabri* (*p* < 0.01), *Q. glauca* (*p* < 0.001), and *Q. spinosa* (*p* < 0.001) showed varying degrees of significance in fiber length comparisons between phloem and xylem. Further analysis using Kruskal–Wallis tests on both wood fiber lengths and bark fiber lengths across the 10 groups also demonstrated highly significant differences (*p* < 0.001) (Figure 10). The detailed measurement and analysis data are presented in Table S5.

### 2.5. Periderm

Periderm, which consists of phellem (cork), phellogen (cork cambium), and phelloderm, functions as a secondary protective tissue replacing the epidermis in stems and roots, and is rarely present in other plant organs. Phellogen undergoes periclinal cell divisions to produce phellem towards the outside and phelloderm towards the inside.

**Figure 11:**
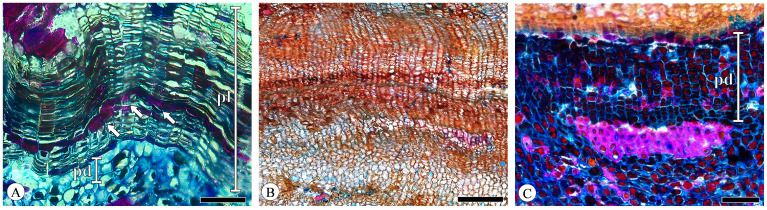
Periderm, transverse section. (**A**) Phellem (pl) and phelloderm (pd), phellem cells’ thickening and lignification (arrows), *Q. aliena*; (**B**) seasonal growth increment of periderm, *Q. acutissima*; (**C**) phelloderm (pd), *Q. variabilis*. Scale bar for (**A**,**C**) = 100 μm; (**B**) = 200 μm.

The periderm displayed a slight curvature, forming discontinuous reticulate (net-like) layers in the transverse section. The phellem cells are radially flattened with thin, uniform cell walls, arranged in a regular and parallel manner without intercellular spaces, typically comprising 10–30 layers (Figure 11A) but potentially extending to dozens or even hundreds of layers in *Quercus acutissima* (Figure 11B) and *Q. variabilis*. The phelloderm cells are difficult to distinguish from the surrounding parenchyma cells, and identification is based on the phelloderm cells’ more regular and distinct radial arrangement. The phelloderm typically consists of from two to five layers of thin-walled cells of equal diameter, with *Q. variabilis* having up to ten or more layers (Figure 11C). In the younger bark, the phellem cells have tannin-filled lumens. The thickening and lignification of the phellem cells are present to a greater or lesser extent in the periderm, distributed in scattered or continuous bands (1 to 3 cell layers; Figure 11A). This phenomenon has also been documented in *Q. cerris* var. *cerris* [31]. No lenticular channels were observed.

The periderm of *Q. acutissima* and *Q. variabilis* exhibits a pronounced seasonal growth increment, with a distinct layering pattern in the phellem (Figure 11B). This is attributed to the larger size and thinner walls of phellem cells (early cork) on the outer side adjacent to the cork cambium, while phellem cells (late cork) closer to the pith side exhibit reduced diameters. Similar patterns were observed in *Q. cerris* var. *cerris* [31,32]. No crystals were found in the periderm.

### 2.6. Lenticels

**Figure 12:**
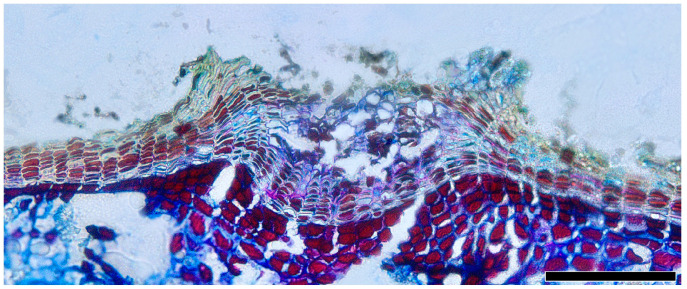
Lenticels, *Q. spinosa*, transverse section. Scale bar = 100 μm.

Lenticels, small pores or openings, are commonly found on the surfaces of plant stems, branches, and bark. Distinguished from the phellem by their intercellular spaces, they play a vital role in facilitating gas exchange between the internal tissues of the plant and the external environment. The lenticels are not always readily observable. The filling tissue of lenticels in *Quercus* is nonstratified (homogeneous) and largely nonsuberized (Figure 12).

### 2.7. IAWA Code

The IAWA list [1] provides standardized terminology and coding for bark anatomy, enabling researchers to consistently describe bark anatomical structures using uniform terminology, thus preventing confusion and ambiguity. This standardization facilitates the comparison of anatomical features across different tree species, aiding researchers in understanding both the similarities and differences between various species while providing a crucial foundation of data for plant taxonomy and evolutionary studies. The results of the IAWA code identification for 10 *Quercus* species based on bark anatomical analyses are presented in Table 1. Detailed micrographs of the bark can be found in Figure S2.

## 3. Discussion

The highly specialized sieve areas located on the terminal walls of sieve tube elements constitute sieve plates. In the 10 *Quercus* species, all types of sieve plates are compound and inclined, with simple sieve plates being observed occasionally. The presence of compound sieve plates is generally associated with longer and more inclined cell end walls, whereas simple sieve plates coincide with relatively horizontal end walls. The evolutionary trend of the phloem was deduced from extensive observations of numerous plant families, in conjunction with the concept that the evolution of phloem and xylem exhibits a parallel trajectory. In angiosperms, the evolution of sieve tube elements is thought to follow a trend from longer to shorter lengths, and from inclined to horizontal sieve plates. This progression also involves a shift from multiple sieve areas in compound sieve plates to single sieve areas in simple sieve plates, ultimately leading to the development of more efficient conduction cells over time [4,33,34,35].

However, research on specific angiosperm families has demonstrated that these two types of sieve plates evolved independently on numerous occasions; that is, there was a convergent evolution of this characteristic [36,37]. These studies utilize data from the Angiosperm Phylogeny Website [38] and specific family phylogeny publications to reconstruct the ancestral states of various anatomical features on phylogenetic trees, with the aim of identifying transitions in systematic evolution. Within the Fagaceae, the simple sieve plates evolved from ancestors with compound sieve plates [36]. In contrast, *Quercus* species predominantly exhibit compound sieve plates, suggesting that *Quercus* may have occupied a conservative position in evolution and possess more relatively primitive features. The presence of a small number of simple sieve plates may suggest that the *Quercus* is not entirely conservative and exhibits certain evolutionary traits.

The length of vessel elements is commonly employed as a metric for assessing the extent of secondary xylem evolution. However, changes in sieve tube element length lack the level of precision observed in vessel elements. During the evolution of woody dicotyledons, the length of fusiform cambial initials has gradually decreased. Consequently, shorter fusiform cambium cells give rise to shorter vessel elements medially. In the phloem, derivatives of fusiform cambium may undergo further division, resulting in a significant shortening of sieve element lengths [39]. Among the 10 studied *Quercus* species, the lengths of the sieve tube elements were either equal to or less than those of vessel elements, thus corroborating this perspective.

When assessing whether companion cells are more primitive or evolved in the evolutionary status of sieve tubes, companion cells are generally not considered as an additional feature for judgment. The observed correlation between companion cells and sieve tube element types in this study aligns with the conclusions of Chavan et al. [40], Esau et al. [41], Cheadle and Esau [42], and Esau [4]. Specifically, the number of companion cells associated with each sieve tube element is positively correlated with the length of the sieve tube elements; longer sieve tube elements may have more associated companion cells. This pattern is exemplified in a single species studied here, where shorter sieve tube elements may have only two associated companion cells, while longer ones may have up to seven. Furthermore, it is suggested that E-type companion cells and short sieve tube elements are highly evolved in terms of their phylogeny, whereas R-type companion cells and long sieve tube elements are primitive. According to the classification of Chavan et al. [40], the companion cell types in this study are all R-type, and the sieve tube types largely conform to Type III, showing a certain degree of correlation between the two. The primitive evolutionary status of R-type companion cells is consistent with the evolutionary status expressed by scalariform sieve plates.

The boundary between the conducting phloem and the nonconducting phloem is not well-defined, and the collapse of sieve tubes has already occurred in the conducting phloem. While the width of the conducting phloem shows seasonal variations, overall, it is relatively narrow in *Quercus* species, often exhibiting collapsed or sieve tubes lacking companion cells near the cambium.

The phloem rays and xylem rays are continuous, since both originate from the same ray initials in the cambium. Consequently, the phloem rays and xylem rays near the cambium often exhibit similarities in terms of height, width, and ray cell composition. However, their identification is limited by certain factors. In the outer part of the phloem, rays may widen, i.e., undergo ray dilatation. Phloem rays located in the conducting phloem, close to the inner side of the cambium, typically exhibit character states that are consistent with those of the xylem and stable in comparison with them. Nevertheless, in certain species, the width of the conducting phloem can be extremely limited and influenced by the course of rays. When the rays display an undulated or wavy course, it becomes demanding to discern the complete ray composition. At times, the course of the rays may be straight, but there could be an overall shift in their direction, thereby increasing the difficulty of slicing radial sections. Therefore, in this study, when identifying ray composition in certain species, the status of the xylem was also referenced.

In *Quercus* broad rays, dilatation commonly occurs, while uniseriate rays experience little to no dilation. This phenomenon may be related to intercellular spaces and often occurs in woody plants with multiseriate rays [1,4]. According to Strasburger [43], in *Tilia*, only the wider rays with intercellular spaces undergo dilation. Some of the uniseriate rays lack intercellular spaces and become crushed in the older phloem.

The tangential bands of sclerenchyma potentially function as a mechanical protective barrier, guarding against the collapse of conducting phloem cells such as parenchyma and sieve tubes [44]. The tangential bands of fibers may also signify increments in growth, although within the phloem, multiple bands may form within each annual growth increment, or broader bands may serve as markers [1]. Further research is warranted to validate the utility of *Quercus* species’ fiber bands in assessing phloem growth increments.

The morphology, origin, and structure of sclerenchyma cells exhibit significant variation, and different types of sclerenchyma cells may possibly be mistaken for each other. Typical sclerenchyma cells, namely fibers and sclereids, are relatively straightforward. In spite of this, the challenge of categorizing a series of forms that blend into one another is widely acknowledged, and precise criteria for distinguishing between these forms have yet to be established [4]. Fiber-sclereids were not found in this study, but they have been recorded in *Q. cerris* var. *cerris* [32].

In numerous instances, categorizing cells into fibers and fiber-sclereids solely based on the timing of their maturation may seem arbitrary. A more practical approach would be to utilize morphological characteristics for cell categorization and to employ the term fiber in a broader sense. The term fiber-sclereid should be used cautiously, only when it challenging to classify the cell type into either the broad category of fiber or that of sclereid [4]. Holdheide [45] emphasized that true fibers are consistently accompanied by crystal-containing sclerified cells, originating from fusiform cells resembling fiber primordia, but are subsequently subdivided into small cells, each containing a crystal.

G-fibers are commonly found in the tissues and organs of angiosperms, including the cortical region, pericyclic region, primary phloem, secondary phloem, secondary xylem, roots, and tendrils [46]. The most distinctive characteristic of tension wood is the existence of G-fibers within the xylem. The identification of tension wood without a microscopic analysis of wood sections is challenging and can even be unfeasible. Thus, the occurrence of G-fibers serves as a fundamental criterion for identifying tension wood. There is limited evidence supporting the association between G-fibers in the phloem and the reaction wood. In gymnosperms, Tomlinson [47] demonstrated that G-fibers in the bark of *Gnetum gnemon* serve similar functions to reaction wood fibers.

Movements permeate all aspects of plant behavior, encompassing both turgor movements and growth movements, although they are not always restricted to the movement of the entire organism [48]. The movement of plants has traditionally been attributed solely to the G-fibers within the xylem, but research indicates that G-fibers located outside the xylem also play a significant role [49]. Lehnebach et al. [50] demonstrated that phloem G-fibers, organized in a trellis network, exhibit higher tensile stress compared to species with a conventional fiber phloem trellis network. In addition, in climbing plants, G-fibers are predominantly present in the tissue responsible for movement [49,51]. Up to now, there have been no genomic and transcriptomic studies conducted on climbing plants’ G-fibers. Further research could investigate the connections between G-fibers, hormones, and the cytoskeleton of tendrils and twining stems [46].

The presence of mechanical tissue and the type of sieve tube appear to be unrelated, and there is a lack of evidence supporting a correlation in their evolution [34,36,52]. Nevertheless, the abundant distribution of sclerenchyma cells in a band-like arrangement provides mechanical support for the bark, which may account for the minimal collapse of sieve tubes. This phenomenon is also observed in the Bignoniaceae, and is evident in species with a lower sclerenchyma content, where a noticeable collapse of sieve tubes occurs [53].

The thickness of the periderm, primarily determined by the number of phellem cell layers, is influenced by precipitation and temperature [54]. In general, it mainly depends on the meristematic activity of the phellogen. In *Q. variabilis*, the phellem cells can reach several hundred radially [55]. Although *Q. cerris* var. cerris [32] and *Q. acutissima* also exhibit significant seasonal increments, they do not achieve such thickness.

The lenticular channels that traverse the cork tissue radially from phellogen to the exterior are intrinsic biological features of the periderm, serving a vital function in facilitating gas exchange [28]. Upon an examination of the transverse sections in this study, no lenticular channels were observed, likely due to their low content. Consequently, in terms of porosity, the *Q. variabilis* cork sample utilized in this study exhibits relatively high quality.

Calcium oxalate (CaC_2_O_4_) is ubiquitous among all major categories of photosynthetic organisms [56]. Calcium oxalate crystals are the predominant crystal type in plant tissues, with prismatic and druse morphologies being the most prevalent. The accumulation of crystals is one of the characteristics of the transition from conducting to nonconducting phloem. Crystals can be deposited in a single tissue or multiple tissues as rays, axial parenchyma, and sclerenchyma. Within a given species, the distribution and morphology of crystals remain relatively constant and have been utilized as a taxonomic characteristic [57,58,59], indicating precise regulation by biological genetic mechanisms [56].

Considering the diverse morphological manifestations and spatial distribution of crystals within plant tissues, numerous hypotheses have been proposed to elucidate their functional significance. These hypotheses encompass mechanisms involving calcium homeostasis, plant defense strategies, detoxification processes including the sequestration of heavy metals or the storage of oxalic acid, cellular ion balance, the provision of structural support, as well as potential optical functions like light gathering and reflection. While certain conjectured functionalities lack empirical validation, an expanding body of research increasingly corroborates their roles in modulating calcium levels, bolstering plant defenses, facilitating detoxification mechanisms, and promoting metal detoxification [56,60,61,62].

Strikingly, it is within maceration material that the morphology of sieve plates in the majority of sieve tubes, along with the count of companion cells associated with them, was observed. The occurrence of simple sieve plates was extremely rare, and they were very small and nearly horizontal, rendering them difficult to observe in sections. While this phenomenon has not been documented in prior studies of *Quercus* bark anatomy, simple sieve plates were consistently observed across all 10 species examined in this study. The quantification of the associated companion cells also posed challenges when observing the sections but was more distinctly identifiable within the maceration material. Limited information regarding the quantity of companion cells has been reported in previous studies. Therefore, it is strongly recommended to observe the morphology of sieve plates and the types of companion cells through maceration.

## 4. Materials and Methods

### 4.1. Sample Collection

The bark samples of 10 *Quercus* species were collected from two locations in Yunnan Province: Xundian County, Kunming City (*Quercus aliena*—four samples, *Q. glauca*—three samples, *Q. spinosa*—three samples, *Q. variabilis*—five samples), and Chaoyang District, Zhaotong City (*Q. aliena* var. *acutiserrata*—three samples, *Q. fabri*—four samples, *Q. aquifolioides*—three samples, *Q. guyavifolia*—three samples, *Q. monimotricha*—four samples, *Q. acutissima*—four samples). Both locations are situated in the warm temperate zone and are characterized by a low-latitude highland monsoon climate with distinct dry and wet seasons. Zhaoyang District has an annual rainfall of 826 mm and an average temperature of 12.1 °C, with an average of 1719.4 h of sunshine per year. Xundian County has an annual rainfall of 1045 mm and an average temperature of 14.4 °C, with an average of 2066.3 h of sunshine per year. Prior to collection, phytomorphology identification was conducted to ensure sample accuracy. Samples’ detailed information is provided in Table S6.

**Figure 13:**
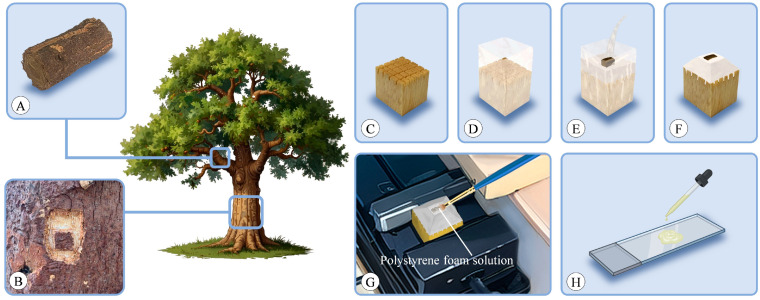
Experimental setup. (**A**,**B**) Sample collection. Bark sourced from perennial branches (**A**) or trunk (**B**). (**C**) Prepared wooden block mold base with a grid-like pattern sawed on the upper surface. (**D**) Mold firmly wrapped with tape. (**E**) Sample placed in the mold and new 100% PEG poured over it. (**F**) After the PEG cooled, it was shaped into a truncated pyramid. (**G**) Slicing facilitated with a polystyrene foam solution. (**H**) Mayer adhesive applied to the slides during mounting.

Bark was typically sourced from the trunk or perennial branches of a tree. Smaller branches can be directly cut using appropriate tools (Figure 13A), while thicker branches and those located on the trunk were collected using the following process. Initially, a small knife was used to outline a square shape on the target tree, followed by a larger square surrounding it. Subsequently, the outer layer of bark was carefully excavated until the underlying xylem was reached. Then, a chisel was utilized to extract the central piece of bark, ensuring that it included some xylem (Figure 13B).

It was crucial to begin with a horizontal section at the top and bottom of the target location, followed by vertical sections on the left and right sides. When using a knife for this purpose, the thorough penetration into the xylem with the knife was ensured. Due to the higher density of xylem compared to phloem, when the knife encounters greater resistance during penetration, this signifies that the xylem was reached. Care must be taken to ensure the correct sequence of creating horizontal and vertical sections, as reversing this order may result in the direct peeling of bark from the cambium layer. Similarly, comparable steps should be applied to excavate the bark’s external outline, starting with the horizontal and then proceeding to the vertical.

### 4.2. Fixation and Conservation

Bark samples were promptly fixed in FAA (formulated as follows: 10% formaldehyde, 5% acetic acid, and 70% ethanol per 100 mL of FAA) after collection [8,9,11]. A vacuum pump was used to treat the samples under suitable negative pressure to eliminate air entrapped within the samples. This step prevents the samples from floating atop the FAA and enhances the permeation of the fixative, thus accelerating the fixation process. Fixation was achieved within 24 h, after which samples could be further preserved or transferred to 70% ethanol. When ready for use, the samples were removed from the solution and immersed in running water for an entire day to prepare for subsequent steps. The addition of a minute quantity of glycerin into the solution acted as a humectant, effectively mitigating desiccation and preserving the specimens’ moisture content over an extended period [63].

### 4.3. Softening

Bark samples were cut into appropriate sizes and placed in wide-neck containers. They were then submerged and sealed in an 8% (concentration can be adjusted between 4–10% based on sample hardness) aqueous solution of ethylenediamine and kept in an oven at 60 °C for 2 days (not exceeding 4 days) [12,64]. During this period, the softening completion could not be precisely determined. However, periodic hardness checks were conducted using a knife, and softening was considered complete when minimal force was required to cut through the samples. The low concentration of ethylenediamine solution is nearly non-toxic and can be reused, although with repeated use, the solution may darken in color [6]. Once softened, the samples were rinsed under running water for several hours in preparation for the embedding.

### 4.4. Embedding

Using PEG 1500 [16] for embedding, the samples were placed in a sealed 10% PEG solution and kept in an oven at 60 °C. The concentration was incrementally increased from 10% to 100% at intervals of 24 h until reaching the final concentration. When the concentration reached 100%, the lid of the container was opened to facilitate the evaporation of excess moisture. In humid environments, PEG 4000 can be added in the final step to reduce moisture absorption after embedding. For embedding in hot environments, PEG 2000 may yield better results.

For each sample, a small wooden block was prepared as a mold base, with a grid-like pattern sawed on its upper surface to increase the area of contact with PEG (Figure 13C). The wooden block was firmly wrapped with tape (Figure 13D), and the sample was placed in the center of the mold, with new 100% PEG poured into the mold (Figure 13E). After the PEG cooled, the tape around the perimeter was removed, and excess PEG on the top was trimmed with a knife until the sample was exposed, with the sides trimmed into a truncated pyramid shape to reduce contact area with the blade during sectioning (Figure 13F). The entire mold containing the wooden block was securely clamped in the holder of the microtome during sectioning to prevent crushing the PEG. The PEG solution could be reused. When the solution’s concentration changed due to excessive use, it could be placed in a wide-neck container and evaporated in an oven to reconfigure to the desired concentration.

### 4.5. Sectioning

The sections were sliced using a sliding microtome (Leica 2000R, Wetzlar, Germany), with thicknesses ranging from 8 to 15 μm for transverse, radial, and tangential sections. Tangential sections were exclusively taken from the conducting phloem. Slicing was facilitated with a polystyrene foam solution (Figure 13G) [6], prepared by dissolving excess polystyrene in butyl acetate. Prior to each slicing procedure, the polystyrene foam solution was applied to the sample surface using a brush, forming a thin film that adhered to the sample. The use of polystyrene foam solution reduces the risk of fragmentation during processes such as sectioning, staining, and mounting.

### 4.6. Staining

The bark typically consists of both lignified and nonlignified cells. Unlike wood, bark anatomical studies generally yield better results with double staining. In this experiment, staining was performed using astra-blue and safranin [65,66]. Initially, the sections were immersed in distilled water to dissolve PEG, followed by staining with a mixture of 0.5% safranin solution and 1% astra-blue solution (adding 2ml of acetic acid per 100 mL of astra-blue solution) for 10 min. Subsequently, excess dye was rinsed off with distilled water. Mayer adhesive [67] was evenly applied to the slides (Figure 13H), which is a mixture of equal amounts of filtered fresh egg white and glycerine. Once the Mayer adhesive became viscous, the stained sections were placed with the side coated with polystyrene facing upwards. The slides were overlapped, separated by impermeable paper, and weighted from the top. After 3 days of standing, slides with attached sections underwent a series of ethanol rinses ranging from 10% to 100% ethanol, with two rinses in 100% ethanol, followed by rinsing in butyl acetate to dissolve the polystyrene. Finally, a little drop of neutral balsam was placed on the section, before sections were closed with a cover glass.

### 4.7. Maceration

Franklin’s method [68] was employed for maceration, in which the xylem and phloem were cut into several centimeter-long strands, placed in small glass tubes, and immersed in a maceration solution prepared from 30% hydrogen peroxide and glacial acetic acid. The tubes were sealed tightly and incubated in a 60 °C oven for 3–6 h. During the maceration process, the samples gradually turned white. Samples were gently pressed with a glass rod; if the samples separated upon pressing, this signified that maceration was finished. The samples were rinsed at least three times, with an additional rinsing after settling at the bottom of the tube. Staining was performed using safranin for xylem cells and astra-blue for phloem cells. To ensure accurate measurement data were obtained for phloem cells and prevent interference from primary fibers, only secondary phloem tissue near the cambium layer was used in phloem samples.

### 4.8. Observation and Measurement

Observations were conducted using an optical microscope (Leica DM 2000 LED). Fluorescence and polarized observations, along with cell measurements, were performed using a biological digital microscope (Nikon ECLIPSE 80i, Tokyo, Japan). Specifically, fiber length was measured at least 100 times, while sieve tube length, sieve tube area, and phloem ray height were measured at least 25 times.

### 4.9. Terminology

The terminology used to describe the microstructure of bark follows Trockenbrodt [69], Richter [70], Evert [3], and the IAWA list of microscopic features for bark [1].

## 5. Conclusions

Anatomical studies of the bark of 10 *Quercus* species reveal similarities in the distribution of tissues, including sieve tube elements and companion cells, axial parenchyma, phloem rays, sclerenchyma, periderm, and rhytidome. This consistency suggests that bark anatomy can be a valuable tool for plant taxonomy, providing supplementary insights beyond traditional classification methods. The presence of inclined compound sieve plates in the phloem of *Quercus* species indicates a potentially conservative evolutionary position within the Fagaceae. Analyzing the morphology, types, and relationships of different cells and tissues enables a better understanding of the evolutionary trends of species or taxa and provides a foundation for subsequent research. These findings aid in species identification and help deepen our understanding of tree physiology and ecology. They also offer important references for the protection and management of plant resources.

Additionally, observations of sieve plate morphology and the number of companion cells in maceration material revealed that single sieve plates, though extremely rare and difficult to observe in sections, were present across all 10 *Quercus* species studied. This finding, not previously documented in *Quercus* bark anatomy, underscores the importance of using maceration material for detailed anatomical studies. The challenges in quantifying companion cells in sections were overcome by maceration, making it a recommended method for the accurate observation of sieve plate and companion cell morphology. This approach provides critical data that enhance our understanding of phloem structure and function in *Quercus* species.

## Figures and Tables

**Table 1:** IAWA codes for 10 *Quercus* species.

Species	IAWA Codes	
	Qualitative Features	Quantitative Features
*Quercus* *acutissima*	1 2 6 8 12 16 21 25 27 31 37 38 41 44 48 49 51 52 58 61 62 64 65 69 71 74 77 79 86 103 105 112 114 115 116 123 129 130 147 148 149 150 174	14: 168–622 μm^2^, 361 ± 109 μm^2^, *n* = 40 15: 131–432 μm, 283 ± 64 μm, *n* = 68 50: 62–321 μm, 157 ± 45 μm, *n* = 104 70: 421–1769 μm, 794 ± 231 μm, *n* = 147
*Q.* *aliena*	1 2 6 8 12 16 21 25 27 31 37 38 41 43 48 49 51 52 61 64 65 69 71 74 77 79 80 84 86 103 105 112 114 116 123 129 130 147 148 149 150 174	14: 67–400 μm^2^, 198 ± 74 μm^2^, *n* = 41 15: 112–362 μm, 226 ± 59 μm, *n* = 88 50: 66–358 μm, 150 ± 48 μm, *n* = 120 70: 345–1574 μm, 872 ± 228 μm, *n* = 203
*Q. aliena* var. *acutiserrata*	1 2 6 8 12 16 21 25 27 31 37 38 41 43 48 49 51 52 61 64 65 69 71 74 77 79 80 84 86 103 105 112 114 116 123 129 130 147 148 149 150 174	14: 87–438 μm^2^, 245 ± 88 μm^2^, *n* = 45 15: 160–382 μm, 275 ± 49 μm, *n* = 55 50: 56–257 μm, 130 ± 38 μm, *n* = 133 70: 459–1233 μm, 766 ± 145 μm, *n* = 118
*Q.* *aquifolioides*	1 2 6 8 12 16 21 25 27 31 37 38 41 43 45 52 58 61 64 65 69 71 74 77 80 84 86 103 105 112 114 116 123 129 130 147 148 149 150 174	14: 110–407 μm^2^, 242 ± 65 μm^2^, *n* = 46 15: 99–315 μm, 222 ± 43 μm, *n* = 135 50: 61–290 μm, 166 ± 47 μm, *n* = 122 70: 382–1238 μm, 673 ± 148 μm, *n* = 145
*Q.* *fabri*	1 2 6 8 12 16 21 25 27 31 37 38 41 43 48 49 51 52 61 64 65 69 71 74 77 79 80 84 86 103 105 112 114 116 123 129 130 147 148 149 150 174 175	14: 69–243 μm^2^, 134 ± 36 μm^2^, *n* = 50 15: 185–374 μm, 277 ± 49 μm, *n* = 36 50: 33–267 μm, 140 ± 48 μm, *n* = 125 70: 301–1607 μm, 708 ± 288 μm, *n* = 140
*Q. glauca*	1 2 6 8 12 16 21 25 27 31 37 38 41 43 48 49 51 52 61 64 65 69 71 74 77 80 84 86 103 105 112 114 115 116 123 129 130 147 148 149 150 174	14: 90–390 μm^2^, 178 ± 52 μm^2^, *n* = 59 15: 191–497 μm, 309 ± 69 μm, *n* = 54 50: 53–371 μm, 144 ± 49 μm, *n* = 113 70: 365–1300 μm, 712 ± 185 μm, *n* = 187
*Q.* *guyavifolia*	1 2 6 8 12 16 21 25 27 31 37 38 41 43 48 49 51 52 61 64 65 69 71 74 77 80 84 86 103 105 112 114 115 116 123 129 130 147 148 149 150 174 175	14: 100–376 μm^2^, 176 ± 56 μm^2^, *n* = 44 15: 162–348 μm, 248 ± 40 μm, *n* = 55 50: 71–325 μm, 145 ± 35 μm, *n* = 132 70: 345–1551 μm, 717 ± 213 μm, *n* = 136
*Q.* *monimotricha*	1 2 6 8 12 16 21 25 27 31 37 38 41 43 48 49 51 52 61 64 65 69 71 74 77 79 80 84 86 103 105 112 114 116 123 129 130 147 148 149 150 175	14: 158–386 μm^2^, 270 ± 51 μm^2^, *n* = 47 15: 129–366 μm, 245 ± 56 μm, *n* = 62 50: 62–253 μm, 146 ± 37 μm, *n* = 116 70: 253–1408 μm, 721 ± 187 μm, *n* = 213
*Q. spinosa*	1 2 6 8 12 16 21 25 27 31 37 38 41 43 48 49 51 52 61 64 65 69 71 74 77 80 84 86 103 105 112 114 116 123 129 130 147 148 149 150 174 175	14: 63–477 μm^2^, 204 ± 83 μm^2^, *n* = 81 15: 163–366 μm, 245 ± 51 μm, *n* = 60 50: 55–375 μm, 146 ± 51 μm, *n* = 161 70: 375–1513 μm, 717 ± 207 μm, *n* = 140
*Q. variabilis*	1 2 6 8 12 16 21 25 27 31 37 38 41 44 48 49 51 52 61 62 64 65 69 71 74 77 79 86 103 105 112 115 116 123 129 130 147 148 149 150 174	14: 448–1370 μm^2^, 867 ± 235 μm^2^, *n* = 46 15: 117–488 μm, 319 ± 78 μm, *n* = 116 50: 90–317 μm, 193 ± 50 μm, *n* = 132 70: 379–1392 μm, 854 ± 225 μm, *n* = 116

## Data Availability

Data recorded in the current study are available in all tables and figures of the manuscript.

## Supplementary Materials

The following supporting information can be downloaded at: https://zenodo.org/records/12068207 (accessed on 3 July 2024).
